# Characterization and regulation of a bacterial sugar phosphatase of the haloalkanoate dehalogenase superfamily, AraL, from *Bacillus subtilis*

**DOI:** 10.1111/j.1742-4658.2011.08177.x

**Published:** 2011-07

**Authors:** Lia M Godinho, Isabel de Sá-Nogueira

**Affiliations:** Centro de Recursos Microbiológicos, Departamento de Ciências da Vida, Faculdade de Ciências e Tecnologia, Universidade Nova de LisboaQuinta da Torre, Caparica, Portugal

**Keywords:** AraL, *Bacillus subtilis*, gene regulation, HAD superfamily (IIA), sugar phosphatase

## Abstract

AraL from *Bacillus subtilis* is a member of the ubiquitous haloalkanoate dehalogenase superfamily. The *araL* gene has been cloned, over-expressed in *Escherichia coli* and its product purified to homogeneity. The enzyme displays phosphatase activity, which is optimal at neutral pH (7.0) and 65 °C. Substrate screening and kinetic analysis showed AraL to have low specificity and catalytic activity towards several sugar phosphates, which are metabolic intermediates of the glycolytic and pentose phosphate pathways. On the basis of substrate specificity and gene context within the arabinose metabolic operon, a putative physiological role of AraL in the detoxification of accidental accumulation of phosphorylated metabolites has been proposed. The ability of AraL to catabolize several related secondary metabolites requires regulation at the genetic level. In the present study, using site-directed mutagenesis, we show that the production of AraL is regulated by a structure in the translation initiation region of the mRNA, which most probably blocks access to the ribosome-binding site, preventing protein synthesis. Members of haloalkanoate dehalogenase subfamily IIA and IIB are characterized by a broad-range and overlapping specificity anticipating the need for regulation at the genetic level. We provide evidence for the existence of a genetic regulatory mechanism controlling the production of AraL.

## Introduction

Phosphoryl group transfer is a widely used signalling transfer mechanism in living organisms, ranging from bacteria to animal cells. Phosphate transfer mechanisms often comprise a part of the strategies used to respond to different external and internal stimuli, and protein degradation [1]. Phosphoryl-transfer reactions, catalysed by phosphatases, remove phosphoryl groups from macromolecules and metabolites [2]. It is estimated that ∼ 35–40% of the bacterial metabolome is composed of phosphorylated metabolites [3]. The majority of cellular enzymes responsible for phosphoryl transfer belong to a rather small set of superfamilies that are all evolutionary distinct, with different structural topologies, although they are almost exclusively restricted to phosphoryl group transfer.

The haloalkanoate dehalogenase (HAD) superfamily is one of the largest and most ubiquitous enzyme families identified to date (∼ 48 000 sequences reported; http://pfam.sanger.ac.uk/clan?acc=CL0137) and it is highly represented in individual cells. The family was named after the archetypal enzyme, haloacid dehalogenase, which was the first family member to be structurally characterized [4,5]. However, it comprises a wide range of HAD-like hydrolases, such as phosphatases (∼ 79%) and ATPases (20%), the majority of which are involved in phosphoryl group transfer to an active site aspartate residue [6–8]. HAD phosphatases are involved in variety of essential biological functions, such as primary and secondary metabolism, maintenance of metabolic pools, housekeeping functions and nutrient uptake [8]. The highly conserved structural core of the HAD enzymes consists of a α-β domain that adopts the topology typical of the Rossmann α/β folds, housing the catalytic site, and is distinguished from all other Rossmanoid folds by two unique structural motifs: an almost complete α-helical turn, named the ‘squiggle’, and a β-hairpin turn, termed the ‘flap’ [6,8,9]. The HAD superfamily can be divided into three generic subfamilies based on the existence and location of a cap domain involved in substrate recognition. Subfamily I possesses a small α-helical bundle cap between motifs I and II; subfamily II displays a cap between the second and third motifs; and subfamily III members present no cap domain [10]. Subfamily IIA, based on the topology of the cap domain, can be further divided into two subclasses: subclass IIA and subclass IIB [10].

Presently, ∼ 2000 sequences are assigned to HAD subfamily IIA, which covers humans and other eukaryotes, as well as Gram-positive and Gram-negative bacteria (http://www.ebi.ac.uk/interpro/IEntry?ac=IPR006357). The *Escherichia coli* NagD [11] and the *Bacillus subtilis* putative product AraL [12] typify this subfamily. NagD is a nucleotide phosphatase, encoded by the *nagD* gene, which is part of the *N*-acetylglucosamine operon (*nagBACD*). The purified enzyme hydrolyzes a number of phosphate containing substrates, and it has a high specificity for nucleotide monophosphates and, in particular, UMP and GMP. The structure of NagD has been determined and the occurrence of *NagD* in the context of the *nagBACD* operon indicated its involvement in the recycling of cell wall metabolites [13]. Although this subfamily is widely distributed, only few members have been characterized.

In the present study, we report the overproduction, purification and characterization of the AraL enzyme from *B. subtilis*. AraL is shown to be a phosphatase displaying activity towards different sugar phosphate substrates. Furthermore, we provide evidence that, in both *E. coli* and *B. subtilis*, production of AraL is regulated by the formation of an mRNA secondary structure, which sequesters the ribosome-binding site and consequently prevents translation. AraL is the first sugar phosphatase belonging to the family of NagD-like phosphatases to be characterized at the level of gene regulation.

## Results and Discussion

### The *araL* gene in the context of the *B. subtilis* genome and *in silico* analysis of AraL

**Fig. 1:**
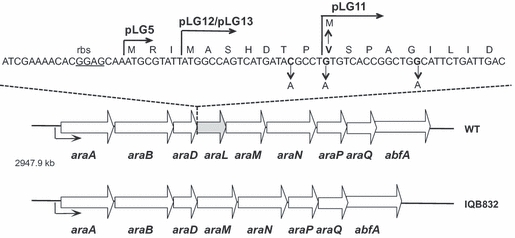
Schematic representation of the *araL* genomic context in *B. subtilis*. White arrows pointing in the direction of transcription represent the genes in the arabinose operon, *araABDLMNPQ-abfA*. The *araL* gene is highlighted in grey and the promoter of the transcriptional unit is depicted by a black arrow. Depicted below the *araABDLMNPQ-abfA* is the in-frame deletion generated by allelic replacement Δ*araL*. Above is displayed the coding sequence of the 5′-end of the *araL* gene. The putative ribosome-binding site, rbs, is underlined. The 5′-end of the *araL* gene present in the different constructs pLG5, pLG11, pLG12 and pLG13, is indicated by an arrow above the sequence. Mutations introduced in the construction of pLG11, pLG13 and pLG26 are indicated below the DNA sequence and the corresponding modification in the primary sequence of AraL is depicted above.

The *araL* gene is the fourth cistron of the transcriptional unit *araABDLMNPQ-abfA* [12]. This operon is mainly regulated at the transcriptional level by induction in the presence of arabinose and repression by the regulator AraR [14,15]. To date, *araL* is the only uncharacterized ORF present in the operon (Fig. 1). The putative product of *araL* displays some similarities to *p*-nitrophenyl phosphate-specific phosphatases from the yeasts *Saccharomyces cerevisiae* and *Schizosaccharomyces pombe* [16,17] and other phosphatases from the HAD superfamily, namely the NagD protein from *E. coli* [13]. Although the yeast enzymes were identified as phosphatases, no biologically relevant substrate could be determined, and both enzymes appeared to be dispensable for vegetative growth and sporulation. The purified NagD hydrolyzes a number of nucleotide and sugar phosphates.

The *araL* gene contains two in-frame ATG codons in close proximity (within 6 bp; Fig. 1). The sequence reported by Sá-Nogueira *et al.* [12] assumed that the second ATG, positioned further downstream (Fig. 1), was the putative start codon for the *araL* gene because its distance relative to the ribosome-binding site is more similar to the mean distance (5–11 bp) observed in *Bacillus* [18]. However, in numerous databases, the upstream ATG is considered as the initiation codon [19]. Assuming that the second ATG is correct, the *araL* gene encodes a protein of 269 amino acids with a molecular mass of 28.9 kDa.

**Fig. 2:**
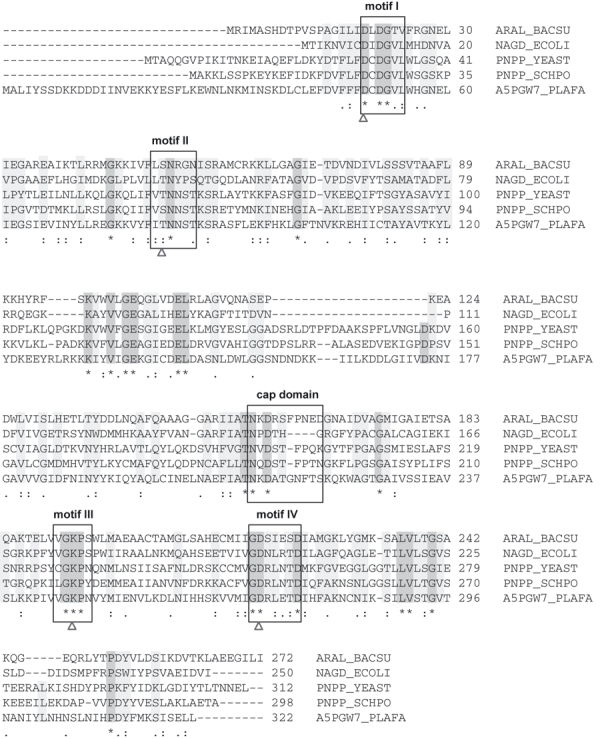
Alignment of AraL with other pNPPases members of the HAD superfamily (subfamily IIA). The amino acid sequences of AraL from *B. subtilis* (P94526), NagD from *E. coli* (P0AF24), the *p*NPPases from *S. cerevisiae* (P19881), *Sz. pombe* (Q00472) and *P. falciparum* (A5PGW7) were aligned using clustal w2 [41]. Similar (‘.’ and ‘:’) and identical (‘*’) amino acids are indicated. Gaps in the amino acid sequences inserted to optimize alignment are indicated by a dash (–). The motifs I, II, III and IV of the HAD superfamily and the cap domain C2 are boxed. Open arrowheads point to the catalytic residues in motifs I–IV. Identical residues in all five sequences, and identical residues in at least three sequences, are highlighted in dark and light grey, respectively.

HAD family members are identified in amino acid alignments by four active site loops that form the mechanistic gear for phosphoryl transfer [8]. The key residues are an aspartate in motif I (D), a serine or threonine motif II (S/T), an arginine or lysine motif III (R/K) and an aspartate or glutamate motif IV (D/E). The NagD family members display a unique α/β cap domain that is involved in substrate recognition, located between motifs II and III [6]. This family is universally spread; however, only a few members have been characterized, such as NagD from *E. coli* [6,11]. NagD members are divided into different subfamilies, such as the AraL subfamily [6], although all proteins present a GDxxxxD motif IV (Fig. 2).

Homologs of the *B. subtilis* AraL protein are found in different species of *Bacteria* and *Archea*, and genes encoding proteins with more than 50% amino acid identity to AraL are present in *Bacillus* and *Geobacillus* species, clustered together with genes involved in arabinose catabolism. An alignment of the primary sequence of AraL with other members of the NagD family from different organisms, namely NagD from *E. coli* (27% identity), the *p*-nitrophenyl phosphatases (pNPPases) from *S. cerevisiae* (24% identity), *Sz. pombe* (30% identity) and *Plasmodium falciparum* (31% identity), highlights the similarities and differences (Fig. 2). AraL displays the conserved key catalytic residues that unify HAD members: the Asp at position 9 (motif I) together with Asp 218 (motif IV) binds the cofactor Mg^2+^, and Ser 52 (motif II) together with Lys 193 (motif III) binds the phosphoryl group (Fig. 2). The cap domain is responsible for substrate binding/specificity; thus, the uniqueness or similarity of the amino acid sequence in this domain may determine enzyme specificity or the lack thereof [10,13,20]. Similar to the other members of the NagD family, AraL shares two Asp residues in the cap domain (Fig. 2). To date, the number of characterized members of this family is scarce. In the present study, we show that AraL possesses activity towards different sugar phosphates. The NagD enzyme was observed to have a nucleotide phosphohydrolase activity coupled with a sugar phosphohydrolase activity [13]. The *P. falciparum* enzyme displayed nucleotide and sugar phosphatase activity together with an ability to dephosphorylate the vitamin B_1_ precursor thiamine monophosphate [21]. The yeast's enzymes are *p*-nitrophenyl phosphatases; however, natural substrates were not found [16,17]. The majority of the enzymes displayed in this alignment show activity to overlapping sugar phosphates [13,21] and it is tempting to speculate that this is related to similarities in the cap domain. On the other hand, the variability and dissimilarity observed in this region may determine the preference for certain substrates (Fig. 2).

### Overproduction and purification of recombinant AraL

Full-length *araL* coding regions, starting at both the first and second putative initiation ATG codons, were separately cloned in the expression vector pET30a(+) (Table 1), which allows the insertion of a His_6_-tag at the C-terminus. The resulting plasmids, pLG5 and pLG12 (Fig. 1), bearing the different versions of the recombinant AraL, respectively, under the control of a T7 promoter, were introduced into *E. coli* BL21(DE3) pLysS (Table 1) for the over-expression of the recombinant proteins. The cells were grown in the presence and absence of the inducer isopropyl thio-β-d-galactoside (IPTG), and soluble and insoluble fractions were prepared as described in the Experimental procedures and analyzed by SDS/PAGE. In both cases, the production of AraL was not detected, although different methodologies for over-expression have been used (see below).

**Table 1:** Plasmids, oligonucleotides, and *E. coli* and *B. subtilis* strains used in the present study. Arrows indicate transformation and point from the donor DNA to the recipient strain. The restriction sites used are underlined, as are single-nucleotide point mutations

Plasmid, strain or oligonucleotide	Relevant construction, genotype or sequence (5′- to 3′)	Source or Reference
Plasmids		
pET30a	Expression vector allowing N- or C-terminal His_6_ tag insertion; T7 promoter, *kan*	Novagen
pMAD	Plasmid used for allelic replacement in Gram-positive bacteria, *bla, erm*	[37]
pAC5	Plasmid used for generation of *lacZ* translational fusions and integration at the *B. subtilis amyE* locus, *bla, cat*	[39]
pLG5	*araL* sequence with the first putative *araL* start codon cloned in the pET30a vector	Present study
pLG10	pMAD derivative with an in frame deletion Δ*araL*	Present study
pLG11	*araL* sequence with mutated GTG codon (valine at position 8) to ATG (methionine) cloned in the pET30a vector	Present study
pLG12	*araL* sequence with the putative second *araL* start codon cloned in the pET30a vector	Present study
pLG13	A pLG12 derivative with a mutation in the *araL* sequence GGC to GAC (Gly12 to Asp)	Present study
pLG25	A pAC5 derivative that contains a translational fusion of *araL* to the *lacZ* gene under the control of the arabinose operon promoter (P*ara*)	Present study
pLG26	A pLG25 derivative with a mutation in the *araL* sequence ACG to AAG (Thr9 to Lys)	Present study
*E. coli* strains		
XL1 blue	(*recA1 endA1 gyrA96 thi-1 hsdr17 supE44 relA1 lac* [F’*proAB lacI*^q^ ZΔM15 Tn*10* (Tetr)]	Stratagene
DH5α	*fhuA2 Δ(argF-lacZ)U169 phoA glnV44 Φ80 Δ(lacZ)M15 gyrA96 recA1 relA1 endA1 thi-1 hsdR17*	Gibco-BRL
BL21(DE3)pLysS	F^−^*ompT hsdS*_B_(r_B_^−^ m_B_^−^) *gal dcm* (DE3) pLysS (Cm^R^)	[40]
*B. subtilis* strains		
168T^+^	Prototroph	[12]
IQB832	Δ*araL*	pLG10→168T^+^
IQB215	Δ*araR*::*km*	[14]
IQB847	*amyE*::P*ara-araL’-’lacZ cat*	pLG25→168T^+^
IQB848	Δ*araR*::*km amyE*::P*ara-araL’-’lacZ cat*	pLG25→IQB215
IQB849	*amyE*::P*ara-araL’* (C→A) *-’lacZ cat*	pLG26→168T^+^
IQB851	*amyE*:: *‘lacZ cat*	pAC5→168T^+^
IQB853	*amyE*::P*ara-araL’* (T→C and C→G) *-’lacZ cat*	pLG27→168T^+^
IQB855	*amyE*::P*ara-araL’* (C→G) *-’lacZ cat*	pLG28→168T^+^
IQB857	*amyE*::P*ara-araL’* (C→A and G→T) *-’lacZ cat*	pLG29→168T^+^
Oligonucleotides		
ARA28	CCTATTGAATTCAAAAGCCGG	
ARA253	TAACCCCAATCTAGACAGTCC	
ARA358	CTGCTGTAATAATGGGTAGAAGG	
ARA439	GGAATTCCATATGCGTATTATGGCCAG	
ARA440	TATTTACTCGAGAATCCCCTCCTCAGC	
ARA444	CGGGATCCACCGTGAAAAAGAAAGAATTGTC	
ARA451	GAATTCATAAAGAAGCTTTGTCTGAAGC	
ARA456	CGGCGCGTCATATGGCCAGTCATGATA	
ARA457	TGATACGCATATGTCACCGGCTGGC	
ARA458	CTCAGCCAATTTGGTTACATCCTTGTCCAAGTCAATCAGAATGCCAGCCGGTGCCAC	
ARA459	GTGTCACCGGCTGGCATTCTGATTGACTTGGACAAGGATGTAACCAAATTGGCTGAG	
ARA460	CGTGAATTCACCGAGCATGTCACCAAAGCC	
ARA477	AATCAGAATGGGATCCGGTGA	
ARA486	CGGCTGACATTCTGATTGACTTGGACGG	
ARA487	CAATCAGAATGTCAGCCGGTGACACAGG	
ARA509	CC AGT CAT GAT AAG CCT GTG TCA CCG	
ARA510	CGG TGA CAC AGG CTT ATC ATG ACT GG	
ARA514	TAATACGCATTTGCTC CGT GTT TTC GTC ATA AAA TAA AAC GCT TTC AAA TAC	
ARA515	GTATTTGAAAGCGTTTTATTTTATGACGAA AAC ACG GAG CAA ATG CGT ATT A	
ARA516	CAC CAC GCT CAT CGA TAA TTT CAC C	
ARA549	GGC CAG TCA TGA TAG GCC TGT GTC ACC	
ARA550	GGT GAC ACA GGC CTA TCA TGA CTG GCC	
ARA551	GCA AAT GCC TAT TAT GGC CAG TCA TGA TAG GCC TGT GTC	
ARA552	GAC ACA GGC CTA TCA TGA CTG GCC ATA ATA GGC ATT TGC	
ARA553	CGG AGC AAA TGC TTA TTA TGG CCA GTC	
ARA554	GAC TGG CCA TAA TAA GCA TTT GCT CCG	

**Fig. 3:**
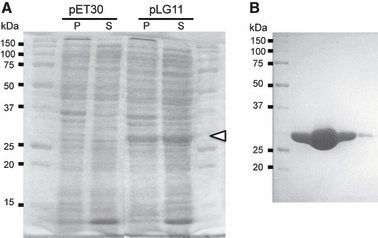
Overproduction and purification of recombinant AraL-His_6_. (A) Analysis of the soluble (S) and insoluble (P) protein fraction (20 μg of total protein) of induced cultures of *E. coli* Bl21(DE3) pLysS harboring pET30a (control) and pLG11 (AraL-His_6_). (B) Analysis of different fractions of purified recombinant AraL eluted with 300 mm imidazole. The proteins were separated by SDS/PAGE 12.5% gels and stained with Coomassie blue. A white arrowhead indicates AraL-His_6_. The size (kDa) of the broad-range molecular mass markers (Bio-Rad Laboratories) is indicated.

On the basis on the alignment of the primary sequence of AraL and NagD, we constructed a truncated version of AraL in pET30a, with a small deletion at the N-terminus (pLG11; Fig. 1). Production of this truncated version of AraL was achieved in *E. coli* BL21 pLys(S) DE3 cells harboring pLG12, after IPTG induction, although the protein was obtained in the insoluble fraction (data not shown). Thus, overproduction was attempted using the auto-induction method described by Studier [22]. In the soluble and insoluble fractions of cells harboring pLG11, a protein of ∼ 29 kDa was detected, which matched the predicted size for the recombinant AraL (Fig. 3A). The protein was purified to more than 95% homogeneity by Ni^2+^-nitrilotriacetic acid agarose affinity chromatography (Fig. 3B).

### Characterization of AraL

**Fig. 4:**
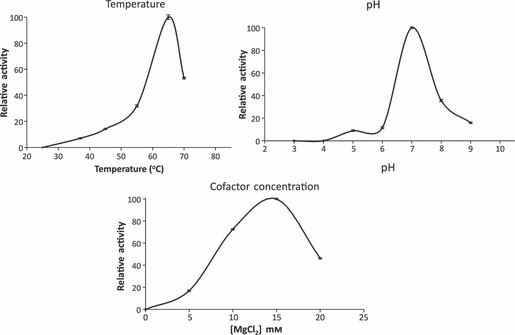
Effect of pH, temperature and co-factor concentration on AraL activity. Enzyme activity was determined using *p*NPP as substrate, at 65 °C, pH 7, and 15 mm MgCl_2_, unless stated otherwise. The results represent the mean of three independent experiments.

AraL phosphatase activity was measured using the synthetic substrate 4-nitrophenyl phosphate (*p*NPP). AraL is characterized as a neutral phosphatase with optimal activity at pH 7 (Fig. 4). Although, at pH 8 and 9, the activity was considerably lower than that observed at pH 7, the values are higher than that observed at pH 6, and no activity was measured below pH 4. The optimal temperature was analyzed over temperatures in the range 25–70 °C. The enzyme was most active at 65 °C and, at 25 °C, no activity was detected (Fig. 4). These biophysical AraL properties fall into the range found for other characterized phosphatases from *B. subtilis*: pH 7–10.5 and 55–65 °C [23–27].

HAD superfamily proteins typically employ a bivalent metal cation in catalysis, and phosphatases, particularly those belonging to the subclass IIA, frequently use Mg^2+^ as a cofactor [3,6,8,13]. The effect of divalent ions (Mg^2+^, Zn^2+^, Mn^2+^, Ni^2+^, Co^2+^) in AraL activity was tested and the results obtained indicated that catalysis absolutely requires the presence of Mg^2+^ (Fig. 4). The addition of EDTA to a reaction containing MgCl_2_, prevented AraL activity (data not shown).

### AraL is a sugar phosphatase

AraL is a phosphatase displaying activity towards the synthetic substrate *p*NPP, although there is no evidence that pNPPase activity is physiologically relevant. The context of *araL* within the arabinose metabolic operon *araABDLMNPQ-abfA*, as involved in the transport of l-arabinose oligomers, further intracellular degradation and catabolism of l-arabinose [12,28,29], suggests a possible role as a phosphatase active towards sugar phosphate intermediates in l-arabinose catabolism, such as d-xylulose 5-phosphate. On the basis of this, as well as the observation that many HAD members display phosphatase activities against various intermediates of the central metabolic pathways, glycolysis and the pentose phosphate pathway [3], we tested AraL activity towards glucose 6-phosphate, fructose 6-phosphate, fructose 1,6-bisphosphate, 3-phosphoglycerate, ribose 5-phosphate, d-xylulose 5-phosphate and galactose 1-phosphate. Although, *B. subtilis* does not utilize d-arabinose, the activity towards d-arabinose 5-phosphate was also assayed. In addition, the nucleotides AMP, ADP, ATP, pyridoxal 5-phosphate and thiamine monophosphate were also screened (Table 2). Although the optimal temperature for enzyme activity is 65 °C, the kinetics parameters were measured at 37 °C, which is the optimal growth temperature for *B. subtilis*. It is noteworthy that, under these conditions, the *K*_M_ determined for *p*NPP is 50 mm (Table 2) compared to 3 mm obtained at 65 °C (data not shown).

**Table 2:** Kinetic constants for AraL against various substrates. Assays were performed at pH 7 and 37 °C, as described in the Experimental procedures. The results are the mean ± SD of triplicates. Substrates tested for which no activity was detected were: ATP, ADP, AMP, ribose 5-phosphate, glycerol 3-phosphate, pyridoxal 5-phosphate and thiamine monophosphate

Substrate	*K*_M_ (mm)	*k*_cat_ (s^−1^)	*k*_cat_/*K*_M_ (s^−1^·m^−1^)
d-xylulose 5-phosphate	29.14 ± 4.87	2.75 ± 0.26	0.943 × 10^2^
Glucose 6-phosphate	24.96 ± 4.08	2.49 ± 0.26	0.998 × 10^2^
d-Arabinose 5-phosphate	27.36 ± 1.8	2.92 ± 0.10	1.06 × 10^2^
Fructose 6-phosphate	34.89 ± 4.51	2.817 ± 0.22	0.807 × 10^2^
Fructose 1,6-bisphosphate	40.78 ± 11.40	1.49 ± 0.26	0.365 × 10^2^
Galactose 1-phosphate	40.74 ± 6.03	4.28 ± 0.40	1.02 × 10^2^
*p*NPP	50.00 ± 23.32	0.012 ± 0.0006	0.24

The AraL enzyme showed reactivity with d-xylulose 5-phosphate, d-arabinose 5-phosphate, galactose 1-phosphate, glucose 6-phosphate, fructose 6-phosphate and fructose 1,6-bisphosphate (Table 2). The *K*_M_ values are high (∼ 30 mm) and above the range of the known bacterial physiological concentrations. In *E. coli*, the intracellular concentration of ribose 5-phosphate, glucose 6-phosphate, fructose 6-phosphate and fructose 1,6-bisphosphate is in the range 0.18–6 mm [3] and, in *B. subtilis*, the measured concentration of fructose 1,6-bisphosphate when cells were grown in the presence of different carbon sources, including arabinose, varies in the range 1.8–14.1 mm [30]. However, we cannot rule them out as feasible physiological substrates because, under certain conditions, the intracellular concentrations of glucose 6-phosphate, fructose 6-phosphate and fructose 1,6-bisphosphate may reach 20–50 mm, as reported for *Lactococcus lactis* [31]. Nevertheless, the mean value of the substrate specificity constant *k*_cat_/*K*_M_ is low (1 × 10^2^ m^−1^·s^−1^); thus, the ability of AraL to distinguish between these sugar phosphate substrates will be limited. The results obtained for AraL are comparable to those obtained for other members of HAD from subfamilies IIA and IIB, which have in common a low substrate specificity and catalytic efficiencies (*k*_cat_/*K*_M_ < 1 × 10^5^ m^−1^·s^−1^) and lack defined boundaries of physiological substrates [10,13]. These features are indicative of enzymes functioning in secondary metabolic pathways.

### Production of AraL in *E. coli* is subjected to regulation

**Fig. 5:**
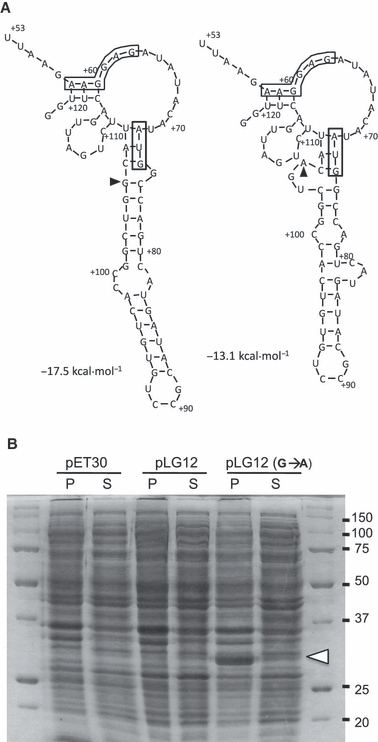
Site-directed mutagenesis at the 5′-end of the *araL* gene and overproduction of recombinant AraL-His_6_. (A) The secondary structure of the *araL* mRNA in pLG12 (left) and pLG13 (right), which bears a single nucleotide change. An arrowhead highlights the mutated nucleotide located at the beginning of the *araL* coding region. The ribosome-binding site, rbs, and the initiation codon (ATG) are boxed. The position relative to the transcription start site is indicated. The free energy of the two secondary structures, calculated by dnasis, version 3.7 (Hitachi Software Engineering Co. Ltd, Tokyo, Japan), is shown. (B) Overproduction of recombinant AraL-His_6_. Analysis of the soluble (S) and insoluble (P) protein fraction (20 μg of total protein) of induced cultures of *E. coli* Bl21(DE3) pLysS harboring pLG12 (AraL-His_6_) and pLG113 (AraL-His_6_ G→A). The proteins were separated by SDS/PAGE 12.5% gels and stained with Coomassie blue. A white arrowhead indicates AraL-His_6_. The sizes (kDa) of the broad-range molecular mass markers (Bio-Rad Laboratories) are indicated.

*In silico* DNA sequence analysis of pLG12 and pLG5 detected the possible formation, in both plasmids, of a mRNA secondary structure, which sequesters the ribosome-binding site. Both, hairpin structures, display a low free energy of −17.5 kcal·mol^−1^ (Fig. 5A) and −22.7 kcal·mol^−1^ (data not shown), respectively, and could impair translation that prevents the production of AraL observed in these constructs (see above). In plasmid pLG11 carrying the truncated version of AraL, overproduction was successful (Fig. 3). Deletion of the 5′-end of the *araL* gene caused an increase of the free energy of the putative mRNA secondary structure (−11.8 kcal·mol^−1^; data not shown). To test the potential involvement of the mRNA secondary structure in the lack of production of the recombinant AraL versions constructed in plasmids pLG12 and pLG5, site-directed mutagenesis was performed using pLG12 as template. A single-base substitution G→A introduced at the 5′-end of the gene (Fig. 1) was designed to increase the free energy of the mRNA secondary structure in the resulting plasmid pLG13. This point mutation increased the free energy from −17.5 kcal·mol^−1^ to −13.1 kcal·mol^−1^ (Fig. 5A). In addition, this modification caused the substitution of a glycine to an aspartate at position 12 in AraL (G12→D; Fig. 1); however, based on the structure of NagD from *E. coli* [13], this amino acid substitution close to the N-terminus is not expected to cause major interference in the overall protein folding. Cell extracts of induced *E. coli* Bl21 pLys(S) DE3 cells carrying pLG13 were tested for the presence of AraL. A strong band with an estimated size of ∼ 29 kDa was detected (Fig. 5B), strongly suggesting that recombinant AraL is produced in *E. coli* when the mRNA secondary structure is destabilized. This observation indicates that the production of AraL is modulated by a secondary mRNA structure placed at the 5′-end of the *araL* gene.

### Regulation and putative role of AraL in *B. subtilis*

**Fig. 6:**
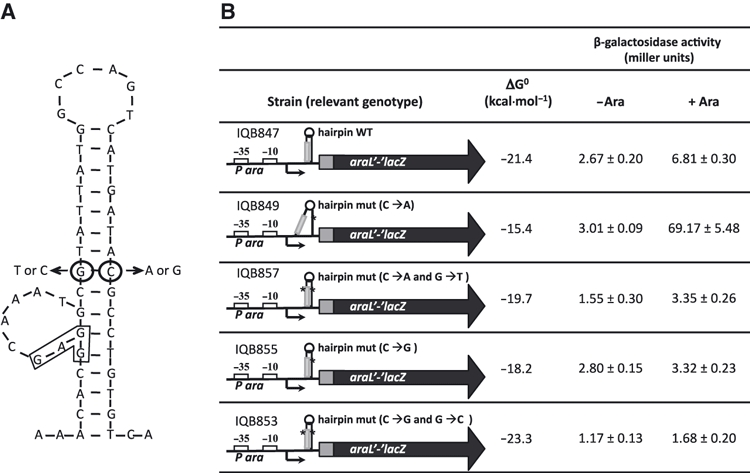
Regulation of *araL* in *B. subtilis*. (A) Site-directed mutagenesis at the 5′-end of the *araL* gene. The secondary structure of the *araABDLMNPQ-abfA* mRNA at the 5′-end of the *araL* coding region is depicted. An arrow highlights the mutated nucleotide (circled) located at the beginning of the *araL* coding region. The ribosome-binding site, rbs, is boxed. (B) Expression from the wild-type and mutant araL′-′lacZ translational fusions. The *B. subtilis* strains IQB847 (P*ara-araL*′*-lacZ*), IQB849 [P*ara-araL*′ (C→A)*-*′*lacZ*], IQB857 [P*ara-araL*′ (C→A and G→T)*-*′*lacZ*], IQB855 [P*ara-araL*′ (C→G)*-*′*lacZ*] and IQB853 [P*ara-araL*′ (C→G and G→C)*-*′*lacZ*] were grown on C minimal medium supplemented with casein hydrolysate in the absence (non-induced) or presence (induced) of arabinose. Samples were analyzed 2 h after induction. The levels of accumulated β-galactosidase activity represent the mean ± SD of three independent experiments, each performed in triplicate. A schematic representation of the translation fusion is depicted and the point mutations in the stem-loop structure are indicated by an asterisk. The free energy of the wild-type (WT) and mutated secondary structures, calculated by dnasis, version 3.7 (Hitachi Software Engineering Co. Ltd), are shown.

In *B. subtilis*, the formation of a similar hairpin structure at the same location is possible and displays a free energy of −21.4 kcal·mol^−1^ (Fig. 6A). To determine its role in the regulation of *araL* expression, a translational fusion of the 5′-end of the *araL* gene to the *lacZ* reporter gene from *E. coli* was constructed and integrated into the *B. subtilis* chromosome, as a single copy, at an ectopic site. The construct comprises the *araL* ribosome-binding site, the initiation codon and a fusion between codon 10 of *araL* and codon 7 of *E. coli lacZ*. The *araL*′*-*′*lacZ* translational fusion is under the control of the strong promoter (P*ara*) of the *araABDLMNPQ-abfA* operon (Fig. 6B). However, expression from the *araL*′*-*′*lacZ* fusion in the presence of arabinose (inducer) is very low, as determined by measuring the levels of accumulated β-galactosidase activity in strain IQB847 (Fig. 6B). By contrast, strain IQB849 carrying a single-base substitution C→A introduced in the hairpin region displayed an augment in *araL*′*-*′*lacZ* expression of ∼ 30-fold in the presence of inducer (Fig. 6B). This point mutation increased the free energy of the mRNA secondary structure from −21.4 kcal·mol^−1^ to −15.4 kcal·mol^−1^ (6 kcal·mol^−1^; Fig. 6B). Furthermore, a double point mutation, C→A and G→T, introduced a compensatory T in the other part of the stem (Fig. 6A), thus regenerating the stem-loop structure in strain IQB857 and drastically reducing the expression of *araL*′*-*′*lacZ* (Fig. 6B). In addition, as described above, a single-point mutation C→G was designed in the same position and the effect was analyzed in strain IQB855 (Fig. 6B). However, no significant effect was detected in the expression of the translational fusion, suggesting that the increase of 3 kcal·mol^−1^ is insufficient for disrupting this particular RNA secondary structure. Similarly, no translation was measured in strain IQB853 carrying a double point mutation, C→G and G→C, which introduced a compensatory C in the other part of the stem (Fig. 6). These results clearly show that the hairpin structure play an active role in the control of *araL* expression. The regulatory mechanism operating in this situation is most probably sequestration of the ribosome binding by the mRNA secondary structure, consequently preventing translation, although the possibility of premature transcription termination by early RNA polymerase release cannot be excluded. Translational attenuation by mRNA secondary structure comprising the initiation region is present in many systems of *Bacteria*, including *B. subtilis* [32]. As a result the nature of the NagD family members displaying low specificity and catalytic activities and lacking clear boundaries defining physiological substrates, regulation at the genetic level was anticipated [13]. In the present study, we show for the first time that a genetic regulatory mechanism controls the expression/production of a member of the NagD family, AraL.

The AraL enzyme encoded by the arabinose metabolic operon *araABDLMNPQ-abfA* was previously shown to be dispensable for arabinose utilization in a strain bearing a large deletion comprising all genes downstream from *araD*. However, this strain displayed some growth defects [12]. To confirm this hypothesis, an in-frame deletion mutation in the *araL* gene was generated by allelic replacement, aiming to minimize the polar effect on the genes of the *araABDLMNPQ-abfA* operon located downstream of *araL* (Fig. 1). The physiological effect of this knockout mutation in *B. subtilis* (strain IQB832 Δ*araL*; Table 1) was assessed by determining the growth kinetics parameters using glucose and arabinose as the sole carbon and energy source. In the presence of glucose and arabinose, the doubling time of the mutant (49.7 ± 0.3 and 52.4 ± 0.1 min, respectively) is comparable to that of the wild-type strain (46.6 ± 0.4 and 52.2 ± 0.5 min, respectively), indicating both the stability of the strain bearing the in-frame deletion and the fact that the AraL enzyme is not involved in l-arabinose utilization. The substrate specificity of AraL points to a biological function within the context of carbohydrate metabolism. The location of the *araL* gene in the arabinose metabolic operon, together with the observation that AraL is active towards d-xylulose 5-phosphate, a metabolite resulting from l-arabinose catabolism, suggests that AraL, similar to other HAD phosphatase members, may help the cell to get rid of phosphorylated metabolites that could accumulate accidentally via stalled pathways. The arabinose operon is under the negative control of the transcription factor AraR and, in an *araR*-null mutant, the expression of the operon is constitutive. In a previous study [14], the addition of arabinose to an early-exponentially growing culture of this mutant resulted in immediate cessation of growth. It was speculated that this effect could be the result of an increased intracellular level of arabinose, which would consequently cause an increase in the concentration of the metabolic sugar phosphate intermediates that are toxic to the cell [14]. Thus, we may hypothesize that AraL possibly plays a role in the dephosphorylation of substrates related to l-arabinose metabolism, namely l-ribulose phosphate and/or d-xylulose phosphate. In addition, because of its capacity to catabolize other related secondary metabolites, this enzyme needs to be regulated. Moreover, the *araL* gene is under the control of the operon promoter, which is a very strong promoter, and basal expression in the absence of inducer is always present [14]. The second level of regulation within the operon that operates in *araL* expression will act to drastically reduce the production of AraL.

## Experimental procedures

### Substrates

*p*NPP was purchased from Apollo Scientific Ltd (Stockport, UK) and d-xylulose 5-phosphate, glucose 6-phosphate, fructose 6-phosphate, fructose 1,6-bisphosphate, ribose 5-phosphate, d-arabinose 5-phosphate, galactose 1-phosphate, glycerol 3-phosphate, pyridoxal 5-phosphate, thiamine monophosphate, ATP, ADP and AMP were obtained from Sigma-Aldrich (St Louis, MO, USA).

### Bacterial strains and growth conditions

*E. coli* strains XL1Blue (Stratagene, La Jolla, CA, USA) or DH5α (Gibco-BRL, Carlsbad, CA, USA) were used for molecular cloning work and *E. coli* BL21 (DE3)(pLysS) was used for the overproduction of AraL (Table 1). *E. coli* strains were grown in LB medium [33] or in auto-induction medium [20]. Ampicillin (100 μg·mL^−1^), chloramphenicol (25 μg·mL^−1^), kanamycin (30 μg·mL^−1^), tetracycline (12 μg·mL^−1^) and IPTG (1 mm) were added as appropriate. *B. subtilis* was grown in liquid LB medium, LB medium solidified with 1.6% (w/v) agar, with chloramphenicol (5 μg·mL^−1^), erythromycin (1 μg·mL^−1^) and X-Gal (50 μg·mL^−1^) being added as appropriate. Growth kinetics parameters of the wild-type and mutant *B. subtilis* strains were determined in CSK liquid minimal medium [34], as described previously [27]. Cultures were grown on an Aquatron® Waterbath rotary shaker (Infors HT, Bottmingen, Switzerland), at 37 °C (unless stated otherwise) and 180 r.p.m., and *A*_600_ was measured in an Ultrospec™ 2100 *pro* UV/Visible Spectrophotometer (GE Healthcare Life Sciences, Uppsala, Sweden).

### DNA manipulation and sequencing

DNA manipulations were carried out as described previously by Sambrook *et al.* [35]. Restriction enzymes were purchased from MBI Fermentas (Vilnius, Lithuania) or New England Biolabs (Hitchin, UK) and used in accordance with the manufacturer's instructions. DNA ligations were performed using T4 DNA Ligase (MBI Fermentas). DNA was eluted from agarose gels with GFX Gel Band Purification kit (GE Healthcare Life Sciences) and plasmids were purified using the Qiagen® Plasmid Midi kit (Qiagen, Hilden, Germany) or QIAprep® Spin Miniprep kit (Qiagen). DNA sequencing was performed with ABI PRIS BigDye Terminator Ready Reaction Cycle Sequencing kit (Applied Biosystems, Carlsbad, CA, USA). PCR amplifications were conducted using high-fidelity Phusion® DNA polymerase from Finnzymes (Espoo, Finland).

### Plasmid constructions

Plasmids pLG5, pLG11 and pLG12 are pET30a derivatives (Table 1), which harbor different versions of *araL* bearing a C-terminal His_6_-tag, under the control of a T7 inducible promoter. The coding sequence of *araL* was amplified by PCR using chromosomal DNA of the wild-type strain *B. subtilis* 168T^+^ as template and different sets of primers. To construct pLG5, oligonucleotides ARA439 and ARA440 (Table 1) were used and introduced unique *Nde*I and *Xho*I restriction sites at the 5′ and 3′ end, respectively, and the resulting PCR product was inserted into pET30a digested with the same restriction enzymes. Using the same procedure, primers ARA457 and ARA440 (Table 1) generated pLG11. ARA457 introduced a mutation, which substitutes Val at position 8 to Met (Fig. 1). Plasmid pLG12 was constructed with primers ARA456 and ARA440. Primer ARA456 inserted an *Nde*I restriction site in the *araL* sequence at the second putative start codon (Fig. 1).

### Site-directed mutagenesis

Vector pLG12 was used as template for site-directed mutagenesis experiments using the mutagenic oligonucleotides set ARA486 and ARA487 (Table 1). This pair of primers generated a G→A substitution at the 5′-end of the *araL* coding region (Fig. 1). This substitution gave rise to a mutation in the residue at position 12 (Gly to Asp) in the resulting plasmid pLG13. PCR was carried out using 1 × Phusion® GC Buffer (Finnzymes), 0.2 μm primers, 200 μm dNTPs, 3% dimethylsulfoxide, 0.4 ng·μL^−1^ pLG12 DNA and 0.02 U·μL^−1^ of Phusion® DNA polymerase in a total volume of 50 μL. The PCR product was digested with 10 U of *Dpn*I, at 37 °C, overnight. The mutation was confirmed by sequencing.

### Overproduction and purification of recombinant AraL proteins in *E. coli*

Small-scale growth of *E. coli* BL21(DE3) pLysS cells harboring pLG5, pLG11, pLG12 and pLG13 was performed to assess the overproduction and solubility of the recombinant proteins. Cells were grown at 37 °C, at 180 r.p.m. and 1 mm IPTG was added when *A*_600_ of 0.6 was reached. Cultures were then grown for an additional 3 h at 37 °C and 180 r.p.m. Whenever protein solubility was not observed, an auto-induction regime for the overproduction of AraL recombinant proteins was used [20]. To prepare the cell-free extracts, the cells were resuspended in lysis buffer (20 mm sodium phosphate buffer, pH 7.4, 62.5 mm NaCl, 10 mm imidazole, glycerol 10%) and disrupted in the presence of lysozyme (1 mg·mL^−1^) by three cycles of freezing in liquid nitrogen and thawing for 5 min at 37 °C, followed by incubation with benzonase nuclease (Novagen®, Darmstadt, Germany). After 15 min of centrifugation at 16 000 ***g*** and 4 °C, the soluble and insoluble fractions of the crude extract were obtained.

For overproduction and purification of recombinant AraL-His_6_, *E. coli* BL21(DE3) pLysS cells harboring pLG11 were grown in 100 mL of auto-induction medium [20]. Cells were harvested by centrifugation at 6000 ***g*** and 4 °C for 10 min. All subsequent steps were carried out at 4 °C. The harvested cells were resuspended in Start Buffer (TrisHCl 100 mm buffer, pH 7.4, 62.5 mm NaCl, 10 mm imidazole, glycerol 10%) and lysed by passing three times through a French pressure cell. The lysate was centrifuged for 1 h at 13 500 ***g*** and the proteins from the supernatant were loaded onto a 1 mL Histrap Ni^2+^-nitrilotriacetic acid affinity column (GE Healthcare Life Sciences). The bound proteins were eluted with a discontinuous imidazole gradient and those fractions containing AraL that were more than 95% pure were dialysed overnight against storage buffer (TrisHCl 100 mm buffer, pH 7.4, 100 mm NaCl, glycerol 10%) and then frozen in liquid nitrogen and kept at −80 °C until further use.

### Protein analysis

Analysis of production, homogeneity and the molecular mass of the enzyme were determined by SDS/PAGE using broad-range molecular weight markers (Bio-Rad Laboratories, Hercules, CA, USA) as standards. The degree of purification was determined by densitometric analysis of Coomassie blue-stained SDS/PAGE gels. The protein content was determined by using Bradford reagent (Bio-Rad Laboratories) with BSA as standard.

### Enzyme assays

#### Phosphatase activity

Phosphatase activity assays were performed using the general substrate *p*NPP. The reaction mixture comprising 100 mm Tris–HCl buffer, pH 7, containing 15 mm MgCl_2_ and appropriately diluted enzyme (20 μg) was incubated at 37 °C for 5 min. Addition of 20 mm*p*NPP started the reaction and the mixture was incubated for an additional 1 h. The reaction was stopped by adding 1 mL of 0.2 m NaOH, the tubes were centrifuged at 16 000 ***g*** for 1 min and 1 mL of the supernatant was recovered for measurement of *A*_405_. A calibration curve for phosphatase activity assays using *p*NPP as a substrate was made using various concentrations (mg·mL^−1^) of *p*-nitrophenol, within the measuring range of the method [36]. Negative controls were made using 20 μg of BSA, and blanks had no protein added. Enzymatic activity was also determined in the presence of 15 mm EDTA, using the same conditions: 1 U of AraL hydrolyses, 1 μmol of substrate per min. Both optimum temperature and pH for enzymatic activity of AraL-His_6_ were determined as described above. The effect of temperature was tested in 100 mm Tris–HCl buffer, pH 7, containing 15 mm MgCl_2_, at temperatures in the range 25–70 °C. The effect of pH on the activity was assayed at 65 °C in a series of Britton–Robinson buffers (0.1 m boric acid, 0.1 m acetic acid and 0.1 m phosphoric acid, pH 3–6, and Tris–HCl buffers, pH 7.0–9.0).

#### Continuous activity assays

All continuous assays were carried out at 37 °C in 100 mm Tris–HCl buffer, pH 7, containing 15 mm MgCl_2_, unless stated otherwise. Glucose production from glucose 6-phosphate was monitored by measurement of the glucose dehydrogenase catalysed reduction of NADP. The initial velocity of glucose formation by dephosphorylation of glucose 6-phosphate in reaction solutions initially containing 20 μg of AraL, 0.7 U of glucose 6-phosphate dehydrogenase, 0.2 mm NADP, 1–15 mmα-glucose 6-phosphate and 15 mm MgCl_2_ in 0.5 mL of 100 mm Tris–HCl (pH 7.5, 37 °C) was determined by monitoring the increase in *A*_340_.

#### Discontinuous assays

Initial phosphate hydrolysis for all substrates used in substrate screening was assessed to detect total phosphate release using the Malachite Green Phosphate Detection Kit (R&D Systems, Minneapolis, MN, USA) in accordance with the manufacturer's instructions. The 150 μL assay mixture comprising 100 mm Tris–HCl buffer (pH 7), containing 15 mm MgCl_2_, was incubated for 1 h at 37 °C. Background phosphate levels were monitored in parallel using a control reaction without the AraL enzyme. *A*_620_ was measured. Steady-state kinetics was carried out using 20 μg of AraL with varying concentrations of substrates. Kinetic parameters were determined using the enzyme kinetics software graphpad prism, version 5.03 (GraphPad Software Inc., San Diego, CA, USA).

### In-frame deletion of araL in *B. subtilis*

To create *B. subtilis* mutant strains with an in-frame deletion of *araL*, plasmid pLG10 was constructed using pMAD (Table 1). Regions immediately upstream and downstream of *araL* were amplified by two independent PCR experiments, from chromosomal DNA of *B. subtilis* 168T^+^, using primers ARA444 and ARA458 (PCR1) and ARA459 and ARA460 (PCR2). The products were joined by overlapping PCR, with primers ARA444 and ARA460 (Table 1), and the resulting 1262 bp fragment was digested with *Bam*HI and *Eco*RI and cloned into pMAD *Bam*HI-*Eco*RI, yielding pLG10. This plasmid harboring an in-frame deletion of *araL* was used for integration and generation of a clean deletion in the *B. subtilis* chromosome, as described previously by Arnaud *et al.* [37]. The in-frame deletion was then confirmed by DNA sequencing and the resulting strain was named IQB832. Transformation of *B. subtilis* was performed as described previously by Anagnostopoulos & Spizizen [38].

### Construction of an-inframe araL′-′lacZ fusion and integration at an ectopic site

To construct plamid pLG25, the arabinose operon promoter region (−81 to +129, relative to the transcriptional start site) was amplified from chromosomal DNA of the *B. subtilis* wild-type strain 168T^+^ using oligonucleotides ARA28 and ARA451 (Table 1). The primers introduced unique *Eco*RI and *Hind*III restriction sites and the resulting fragment was sub-cloned into the same sites of the cloning vector pLG1 (L. M. Godinho & I. de Sá Nogueira, unpublished results). Sequentially, the 5′-end of the *araL* coding region comprising the rbs (position +3910 to +4020, relative to the transcriptional start site of the operon) was amplified from the wild-type strain with oligonucleotides ARA253 and ARA477 (Table 1), which carry unique *Xba*I and *Bam*HI restriction sites and allow the insertion of this fragment between the *Nhe*I and *Bam*HI sites of pLG1. In the resulting plasmid, a deletion of the *araA* rbs and *araA* start site present in the arabinose promoter region (P*ara*) was performed by overlapping PCR using two set of primers: ARA358 and ARA514, and ARA515 and ARA516 (Table 1). The resulting fragment of 216 bp, comprising the arabinose promoter region (P*ara*) from −81 to +80 fused to the 5′-end of the *araL* coding region from +3952 to +4007, was inserted into the vector pAC5 (Table 1), yielding pLG25. Plasmid pLG25 carries a translational fusion between codon 10 of *araL* and codon 7 of *E. coli lacZ*. pLG25 was used as template for site-directed mutagenesis experiments using the mutagenic oligonucleotides set ARA509 and ARA510 (Table 1), as described above. This pair of primers generated a C→A substitution at the 5′-end of the *araL* coding region (Figs 1 and 6A). The substitution gave rise to a mutation in the residue at position 9 (Thr to Lys) in the resulting plasmid pLG26. pLG26 was then used as template for site-directed mutagenesis using primers ARA549 and ARA550, which allowed a C→G substitution (Thr to Arg) in position 9, thus giving rise to pLG28. The oligonucleotide set ARA551 and ARA552 introduced a double point mutation at the 5′-end of the *araL* coding sequence. Using pLG26 as template, the set of primers caused T→C (Arg to Pro) and C→G (Thr to Arg) mutations in the second and ninth positions, respectively, yielding pLG27. Plasmid pLG29 was obtained using site-directed mutagenesis from pLG26, using the oligonucleotide pair ARA553 and ARA554, giving rise to a G→T substitution (Arg to Leu) in position 2. The constructions were confirmed by DNA sequencing.

DNA from plasmids pLG25, pLG26, pLG27, pLG28 and pLG29, carrying the different *araL*′-′*lacZ* translational fusions, was used to transform *B. subtilis* strains (Table 1) and the fusions ectopically integrated into the chromosome via double recombination with the *amyE* gene back and front sequences. This event led to the disruption of the *amyE* locus and was confirmed as described previously [14].

### β-Galactosidase activity assays

Strains of *B. subtilis* harboring the transcriptional *lacZ* fusions were grown were in liquid C minimal medium [14] supplemented with casein hydrolysate 1% (w/v), and arabinose was added to the cultures, when necessary, at a final concentration of 0.4% (w/v), as described previously [14]. Samples of cell culture (100 μL) were collected 2 h (i.e. exponential growth phase) after induction and the level of accumulated β-galactosidase activity was determined by incubation for 30 min at 28 °C with the chromogenic substrate, as described previously [14].

## Abbreviations

HAD: haloalkanoate dehalogenase
IPTG: isopropyl thio-β-d-galactoside
*p*NPP: 4-nitrophenyl phosphate
pNPPase: *p*-nitrophenyl phosphatase
